# Neurocysticercosis: A Challenging Case in New Mexico

**DOI:** 10.7759/cureus.57139

**Published:** 2024-03-28

**Authors:** Brian R Beyer, Simran S Banga, Marshall Caraveo

**Affiliations:** 1 Medicine, Burrell College of Osteopathic Medicine, Las Cruces, USA

**Keywords:** taenia solium, intraparenchymal cyst, brain abscess, taenia solium solitary lesion, neurocysticercosis

## Abstract

Neurocysticercosis, a parasitic infection caused by the larvae of *Taenia solium*, presents a diagnostic challenge, particularly in non-endemic regions. This case report evaluates the intricacies of diagnosing neurocysticercosis in a 62-year-old male presenting with an intractable headache and altered mental status, initially confounded by the absence of an exposure history. Despite lacking typical risk factors, including immunocompromise or HIV infection, his travel history to an endemic area prompted a rigorous clinical work-up. Imaging studies revealed characteristic ring-enhancing lesions indicative of neurocysticercosis, while further serological tests yielded mostly equivocal results. Infectious disease consultation and workup helped confirm a probable diagnosis. Prompt initiation of anti-helminthic therapy led to marked clinical improvement. This case underscores the importance of considering neurocysticercosis in the differential diagnosis of intracranial lesions, especially in regions with Hispanic populations, and advocates for increased awareness and research to enhance timely identification and management, thereby improving patient outcomes.

## Introduction

In the realm of brain abscesses, neurocysticercosis, caused by the larvae of *Taenia solium*, demands a heightened level of suspicion. This tapeworm infection of the central nervous system poses severe consequences, particularly in young adults, and represents a significant proportion of intracranial lesions, especially in the Hispanic population [1]. The condition's presentation varies widely, encompassing symptoms such as acquired epilepsy, hydrocephalus, and headaches, often tied to the lesion's location [2].

Identifying *Taenia solium* in an acute setting is challenging due to the absence of an exposure history. When infected, the tapeworm can reside in the small intestine for years, producing proglottids that can contain up to 100,000 eggs. These eggs are released into the bloodstream, penetrate the intestinal wall, and spread through the blood, ultimately infecting organs such as the brain. Humans serve as definitive hosts, allowing the helminth to reach the central nervous system. Ingestion of undercooked pork or exposure to contaminated water bodies represents common routes of transmission [3].

Delays in diagnosis and treatment are common due to the absence of exposure history to *Taenia solium*, a helminth historically neglected in non-endemic areas [4]. Healthcare providers in regions like the Southwest should maintain heightened suspicion for *Taenia solium* when evaluating new-onset seizures in older adults. Prompt intervention is crucial, requiring anti-helminth therapy to prevent clinical deterioration and achieve resolution of neurocysticercosis.

Despite their clinical significance,* Taenia solium* infections remain underreported in non-endemic areas, emphasizing the need for increased clinical suspicion for an accurate diagnosis. This case report details an instance of neurocysticercosis initially presenting as a solitary ring-enhancing lesion, aiming to guide healthcare providers in considering *Taenia solium* as a viable differential diagnosis for intracranial lesions.

## Case presentation

A 62-year-old male with no significant medical history presented with an intractable headache and altered mental status. History obtained from the patient’s wife revealed recent travels from Mexico. During the drive home, the patient experienced increasing confusion and an occipital headache that persisted for three days.

**Figure 1:**
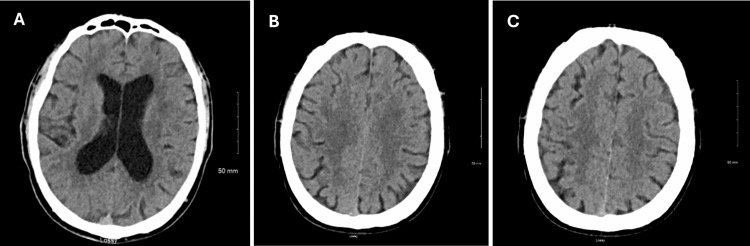
CT scan showing nonspecific changes for evaluation of altered mental status CT: computed tomography

**Figure 2:**
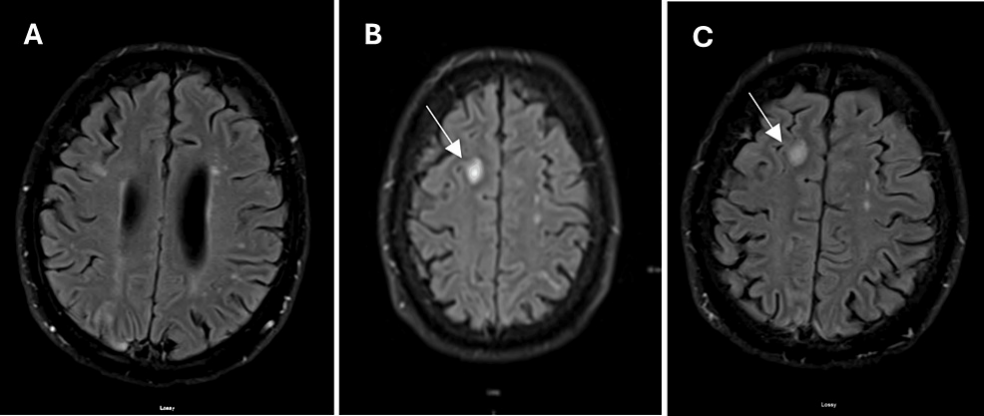
MRI with intravenous contrast showed multiple areas of abnormal enhancement within the brain parenchyma, including a ring-enhancing lesion in the right frontal lobe MRI: magnetic resonance imaging

Upon admission, the patient underwent a CT scan, which showed nonspecific changes on day 1 (Figure 1). This was followed by an MRI, which revealed ring-enhancing lesions in the right frontal lobe and small, intense T2 lesions, suggestive of potential cysticercosis (Figure 2). Given his travel history, confusion, and word-finding difficulty, he was diagnosed with complex partial seizures and a suspected neurocysticercosis infection. Treatment with levetiracetam, albendazole, ceftriaxone, metronidazole, vancomycin, and prednisone resulted in neurological improvement.

Over the next six days, supportive measures were taken, and infectious disease consultation was sought. Additional serological tests revealed mildly elevated cysticercosis serology with elevated protein, positive Fungitell, and negative results for HIV 1/2 antibodies, Coccidioides antibodies, Aspergillus antibodies, venereal disease research laboratory, and cryptococcal antigen.

**Figure 3:**
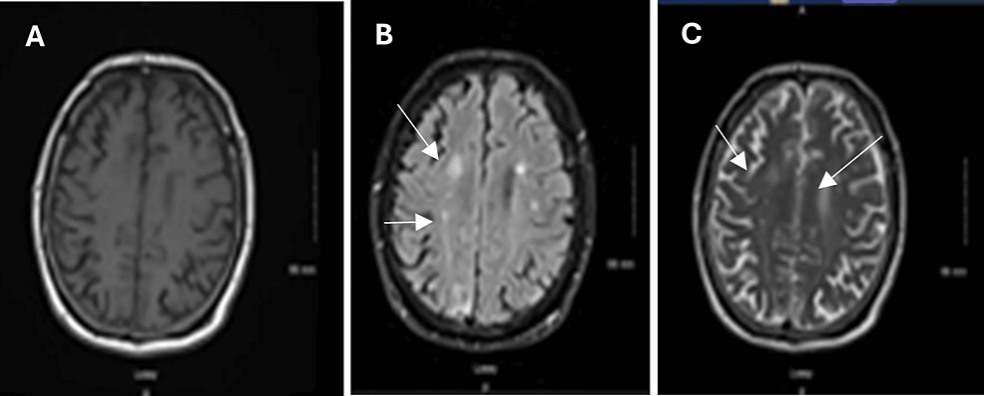
MRI with intravenous contrast shows a near-complete resolution of enhancement. Findings can be indicative of a response to therapy. Normalization of the diffusion signal is observed MRI: magnetic resonance imaging

**Figure 4:**
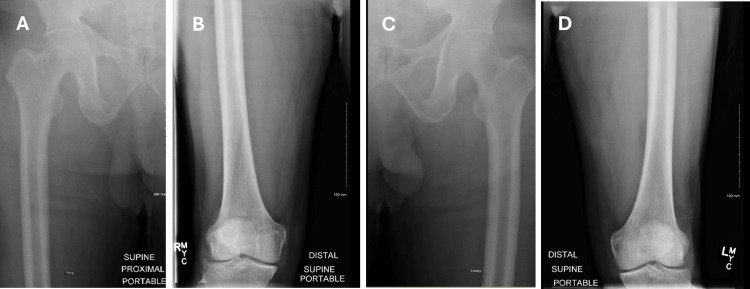
X-ray showing mild to moderate nonspecific degenerative changes of bilateral lower extremities. Images A and B correspond to the right lower extremity. Images C and D correspond to the left lower extremity

**Figure 5:**
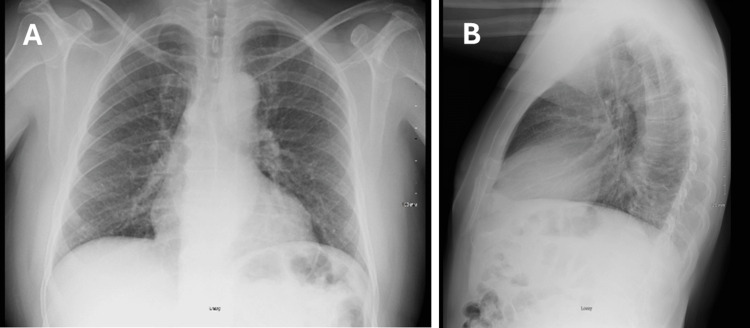
Chest X-ray demonstrating nonspecific interstitial infiltrates

On days 7, 8, and 9, further imaging was conducted to refine the differential diagnosis. Subsequent MRI showed near-complete resolution of ring-enhancing edema with normalization of diffusion (Figure 3). X-rays of the femur revealed no evidence of calcifications in the muscles, complicating the diagnosis of neurocysticercosis (Figure 4). The resolving enhancements on the MRI suggested ruling out potential lymphoproliferative disorders. Extensive work-up for tuberculosis, with possible tuberculoma formation, was undertaken but ruled out based on a follow-up chest X-ray showing nonspecific interstitial prominences without evidence of latent or active infection (Figure 5).

The infectious disease consultant concluded that the patient met one major diagnostic criterion, including resolution of intracranial cystic lesions after therapy with albendazole, and three minor criteria: evidence of lesions compatible with neurocysticercosis on imaging, clinical manifestations suggestive of neurocysticercosis, and positive anticysticercal antibodies. Additionally, he met one epidemiological diagnostic criterion as an individual from an endemic cysticercosis region. These criteria led to a highly probable diagnosis of neurocysticercosis.

The patient’s condition significantly improved, with no further altered mental status. The infectious disease consultant concluded that the final diagnosis was neurocysticercosis/brain abscess, given the clinical presentation and MRI findings. Treatment continued with albendazole, levetiracetam, ceftriaxone, and metronidazole. The patient and his family opted for home management. Discharge instructions included continuing albendazole 400 mg twice daily for 10 days, metronidazole 500 mg three times daily for 21 days, and switching from ceftriaxone to ciprofloxacin 500 mg once daily for 10 days. The patient was advised to follow up for a repeat MRI in three months and to consult infectious disease specialists regarding symptom resolution. Although the formal criteria for neurocysticercosis were not entirely met, the patient responded well to treatment.

## Discussion

When thinking of neurocysticercosis, it is common to see pathology in a patient who is immunocompromised. This was not the case for our patient, who did not have any indication of neutropenia or lymphocytopenia per his blood work. He was also HIV-negative, which is significant considering many reported cases of *Taenia solium* occur in the setting of HIV. Butchers in endemic regions will often cook pork regardless of whether cysts are visibly present, which is detrimental to the consumer since these cysts are not visible after the pork has been cooked [5]. Given the patient’s travel history, endorsement of pork consumption, and socioeconomic conditions, it is likely that his presentation is due to neurocysticercosis.

Neurocysticercosis often presents as incidental findings on CT scans in asymptomatic patients with no prior history of exposure. In our case, the initial clinical symptoms manifested as altered mental status and headaches, which were likely attributed to the mass effect rather than hydrocephalus or focal neurological deficits. Left untreated, this condition carries a high mortality rate and the potential for subsequent neurological deficits [6].

The treatment regimen for neurocysticercosis typically includes albendazole or praziquantel, often augmented with steroids. Workup is time-sensitive because said treatment would not be as efficacious if ring-enhancing lesions progressed to become calcified lesions. Steroids play a crucial role in preventing seizure relapses in patients by reducing inflammation [7]. Interestingly, there are no specific regimens to prevent neurocysticercosis in individuals traveling to endemic areas. Hence, proper food handling and sanitation practices are the primary precautions travelers should observe.

A definitive diagnosis of *Taenia solium* typically relies on CT and/or MRI of the brain, revealing one or multiple ring-enhancing lesions. Diagnosis can also be strongly suggested through an appropriate lumbar puncture, which may detect anti-neurocysticercosis antibodies [8]. However, it's important to note that cerebrospinal fluid analysis is frequently negative or shows nonspecific abnormalities.

## Conclusions

Diagnosing neurocysticercosis can be particularly challenging, especially in cases where patients lack a prior clinical history of exposure to *Taenia solium*. The initial presentation of ring-enhancing lesions highlighted in this report underscores the potentially fatal nature of *Taenia solium* presentations. Emphasizing the need for a comprehensive diagnostic workup cannot be overstated. Healthcare providers should prioritize considering *Taenia solium* in the differential diagnosis of intracranial lesions, especially when presented with specific clinical features indicative of the condition. This case serves as a poignant reminder of the necessity for heightened clinical suspicion and a thorough evaluation, even in non-endemic areas. Early recognition and treatment are pivotal in preventing adverse outcomes.
